# Machine learning for identification of restless legs syndrome-associated factors and classification model development in end-stage renal disease patients

**DOI:** 10.3389/fneur.2026.1836086

**Published:** 2026-04-29

**Authors:** Tao Yuan, Na Sun, Lanbo Teng, Chuhan Xu, Yunyan Wang, Wenyu Zhang, Wenxiu Chang

**Affiliations:** 1 Department of Nephrology, Tianjin First Central Hospital, Tianjin, China; 2 Tianjin Medical University, Tianjin, China; 3 National Clinical Research Center for Chinese Medicine, First Teaching Hospital of Tianjin University of Traditional Chinese Medicine, Tianjin, China

**Keywords:** diagnostic screening, end-stage renal disease, interpretable artificial intelligence, machine learning, restless legs syndrome, SHAP, support vector machine

## Abstract

**Background:**

Restless legs syndrome (RLS) is a common and debilitating complication in end-stage renal disease (ESRD) patients undergoing dialysis, significantly impairing sleep quality and quality of life. Screening of prevalent cases remains challenging. This study aimed to develop and validate an interpretable machine learning-based classification model for identifying RLS status in ESRD patients.

**Methods:**

A total of 396 ESRD patients (173 hemodialysis, 223 peritoneal dialysis) were enrolled from April to October 2024. Patients were randomly divided into training (70%, *n* = 287) and testing (30%, *n* = 109) sets. Feature selection was performed using LASSO regression with five-fold cross-validation, followed by Akaike Information Criterion (AIC) refinement. Nine machine learning algorithms were developed: Logistic Regression (LR), Random Forest (RF), Support Vector Machine (SVM), Gradient Boosting Machine (GBM), K-Nearest Neighbors (KNN), Decision Tree (DT), Artificial Neural Network (ANN), Multivariate Adaptive Regression Splines (MARS), and Quadratic Discriminant Analysis (QDA). Model performance was evaluated using discrimination (AUC-ROC), calibration (Brier score, calibration curves), and clinical utility (Decision Curve Analysis, DCA). SHapley Additive exPlanations (SHAP) was employed to enhance model interpretability.

**Results:**

Five variables were selected: β2-microglobulin, hemoglobin, diabetes mellitus, coronary heart disease, and alcohol consumption. SVM demonstrated optimal performance with AUC of 0.791 (95% CI: 0.702–0.879) in the testing set, outperforming other models. SVM achieved accuracy of 0.761, sensitivity of 0.711, specificity of 0.797, F1-score of 0.711, and Brier score of 0.183. Calibration curves showed good agreement between estimated and observed probabilities. DCA confirmed favorable net clinical benefit across threshold probabilities. SHAP analysis identified β2-microglobulin (mean |SHAP| = 0.131) and anemia as the most influential variables with diabetes, coronary heart disease, and alcohol consumption contributing moderately. SHAP dependence plots revealed interactions between β2-microglobulin and hemoglobin, as well as diabetes modifying the protective effect of higher hemoglobin.

**Conclusion:**

We developed and validated an interpretable SVM-based classification model for identifying RLS in ESRD patients using readily available clinical variables. This model demonstrates promising performance and requires prospective external validation in multi-center cohorts before clinical implementation. This tool may facilitate screening of prevalent RLS cases and inform clinical decision-making.

## Introduction

Restless legs syndrome (RLS), also known as Willis-Ekbom disease, is a chronic sensorimotor neurological disorder characterized by an irresistible urge to move the legs, often accompanied by uncomfortable sensations that worsen during rest and improve with movement (1, 2). The diagnosis requires fulfillment of five essential criteria established by the International Restless Legs Syndrome Study Group (IRLSSG): (1) an urge to move the legs, usually accompanied by uncomfortable sensations; (2) onset or worsening during periods of rest or inactivity; (3) partial or complete relief by movement; (4) worsening or exclusive occurrence in the evening or night; and (5) exclusion of alternative medical or behavioral conditions.

End-stage renal disease (ESRD) represents one of the most prevalent secondary causes of RLS, with reported prevalence ranging from 20% to 65% among dialysis patients-substantially higher than the 5%–15% observed in the general population (3–5). The pathophysiology of RLS in ESRD is multifactorial, involving uremic toxin accumulation, iron deficiency, dopaminergic dysfunction, chronic inflammation, and peripheral neuropathy (6, 7). Importantly, RLS in ESRD patients is associated with severe sleep disruption, depression, anxiety, reduced quality of life, and increased cardiovascular morbidity and mortality (8–10).

Despite its significant clinical impact, RLS remains underdiagnosed in clinical practice, particularly in dialysis populations where symptoms may be attributed to other uremic complications. Screening of prevalent cases could enable targeted interventions, including optimization of dialysis adequacy, iron supplementation, dopaminergic therapy, and lifestyle modifications (11, 12). However, traditional risk stratification approaches relying on single risk factors lack sufficient predictive accuracy for clinical decision-making.

The application of machine learning (ML) prediction models in medical research has expanded rapidly, with studies demonstrating utility in diverse disease contexts including sepsis-induced coagulopathy prediction (13), assessment of tumor recurrence and metastasis (14), and neurological disorder identification (15). These developments highlight the potential for ML-based tools to enhance clinical decision support, while underscoring the importance of rigorous validation and interpretability for clinical adoption. Machine learning also has emerged as a powerful paradigm for developing predictive models in nephrology, offering advantages in handling complex, high-dimensional data and capturing non-linear relationships between variables (16, 17). While several studies have identified risk factors for RLS in ESRD, few have developed and validated comprehensive classification model using ML methodologies (18, 19). Furthermore, the “black box” nature of many ML algorithms has limited their clinical adoption, as clinicians require interpretable models that provide mechanistic insights beyond mere predictions (20).

The advent of explainable artificial intelligence (XAI) techniques, particularly SHapley Additive exPlanations (SHAP), has addressed this limitation by quantifying the contribution of each feature to individual predictions and revealing interaction effects between variables (21, 22). SHAP values enable both global interpretation (feature importance across the entire dataset) and local interpretation (individual patient-level explanations), facilitating clinical translation.

This study aimed to: (1) develop and validate multiple ML-based classification models for identifying RLS in a well-characterized ESRD cohort; (2) identify the optimal model through comprehensive performance evaluation; and (3) apply SHAP analysis to enhance interpretability and elucidate the complex interplay between associated factors.

## Materials and methods

### Study design and population

This prospective cross-sectional study was conducted at Tianjin First Central Hospital, China, from April to October 2024. Consecutive ESRD patients undergoing maintenance dialysis (hemodialysis [HD] or peritoneal dialysis [PD]) were enrolled. Inclusion criteria were: (1) age ≥18 years; (2) dialysis duration ≥3 months; (3) ability to complete standardized questionnaires. Exclusion criteria included: (1) pre-existing RLS diagnosis prior to dialysis initiation; (2) Parkinson’s disease or other movement disorders; (3) cognitive impairment precluding informed consent; (4) peripheral neuropathy from non-uremic causes; (5) lower limb amputation or surgery; (6) acute infection or unstable medical condition; (7) incomplete data.

The study protocol was approved by the Ethics Committee of Tianjin First Central Hospital (approval number: 2024-076). All participants provided written informed consent. The study was conducted in accordance with the Declaration of Helsinki.

### RLS assessment

RLS diagnosis was established through structured interviews conducted by trained nephrologists using the IRLSSG 2014 diagnostic criteria (1). Severity was classified as mild (occasional symptoms, minimal sleep disturbance), moderate (symptoms <2 times weekly, moderate sleep interference), or severe (symptoms ≥3 times weekly, severe sleep disruption with daytime symptoms) (23). Sleep quality was assessed using the Pittsburgh Sleep Quality Index (PSQI), with scores ≥11 indicating sleep disturbance (24).

### Data collection

Comprehensive demographic, clinical, and laboratory data were collected through electronic medical records and standardized interviews. Variables included: age, sex, education level, dialysis modality, dialysis vintage, comorbidities (hypertension, diabetes mellitus, coronary heart disease, cerebrovascular disease), lifestyle factors (smoking, alcohol consumption), medication use, and complete blood count with differential, comprehensive metabolic panel, iron studies, parathyroid hormone, and β2-microglobulin.

For hemodialysis patients, detailed dialysis prescriptions were documented, including session frequency (3 times weekly), duration (4 h per session), dialyzer type (low-flux polysulfone membrane: 57 patients, 32.7%; low-flux triacetate membranes: 51 patients, 29.3%; high-flux polysulfone membrane: 65 patients, 36.1%), blood flow rate (250–300 mL/min), and dialysate flow rate (500 mL/min). Additionally, 23 hemodialysis patients (13.3%) received concomitant hemodiafiltration once every 2 weeks with a substitution volume of 15–20 L per session. For peritoneal dialysis patients, treatment modalities comprised continuous ambulatory peritoneal dialysis (CAPD, 181 patients, 81.1%) and automated peritoneal dialysis (APD, 42 patients, 18.9%), with daily dialysate exchange volumes of 6–8 L.

Missing data were minimal (all variables <5%). To reduce potential bias, missing values were imputed within the training set using median imputation for continuous variables and mode imputation for categorical variables, with the same parameters subsequently applied to the testing set.

### Feature selection

To prevent overfitting and enhance model generalizability, we employed a two-stage feature selection process that combines the strengths of regularization and traditional statistical inference. In the first stage, Least Absolute Shrinkage and Selection Operator (LASSO) regression with five-fold cross-validation was applied to the training set to perform automatic variable selection and regularization, handling high-dimensional data and multicollinearity through L1 regularization, where the optimal regularization parameter (*λ*) was determined using the minimum cross-validation error criterion (lambda.min) with the alpha parameter set to 1 for LASSO regularization, and variables with non-zero coefficients were retained for subsequent analysis. LASSO regression automatically shrinks less important coefficients to zero. In the second stage, these retained variables were entered into a multivariable logistic regression model, and stepwise backward elimination based on the Akaike Information Criterion (AIC) was performed to further refine the model, optimizing model parsimony by removing variables without statistical significance (*p* > 0.05), ensuring that the final variables contribute independently to the outcome. This hybrid approach balances data-driven variable selection with statistical rigor. Variables showing *p* > 0.05 were sequentially removed until all remaining variables achieved statistical significance.

To assess the stability of our feature selection strategy, we performed sensitivity analyses: (1) comparing models using LASSO-only selected variables vs. LASSO+AIC refined variables; (2) evaluating model performance with bootstrap resampling (1,000 iterations) to confirm variable selection consistency. The final 5-variable model showed stable performance across these analyses (AUC range: 0.785–0.791).

### Model development

Nine machine learning algorithms were implemented using R software (version 4.4.2) with specific configurations for each approach. Logistic regression served as the baseline linear classifier using the glm function, while random forest was configured as an ensemble of 500 decision trees with bootstrap aggregation using the randomForest package. Support vector machine employed a kernel-based classifier with radial basis function kernel and cost parameter optimized via grid search using the e1071 package. Gradient boosting machine was set up as a sequential ensemble with 100 trees, interaction depth of 3, and shrinkage of 0.1 using the gbm package. K-nearest neighbors utilized instance-based learning with *k* = 5 and Euclidean distance through the class package. Decision tree applied recursive partitioning with Gini impurity and minimum node size of 10 using the rpart package. Artificial neural network was configured as a multi-layer perceptron with one hidden layer containing 5 neurons, weight decay of 0.001, and maximum iterations of 1,000 using the nnet package. Multivariate adaptive regression splines employed non-linear regression with maximum 2-degree interaction through the earth package. Quadratic discriminant analysis was implemented as a probabilistic classifier with quadratic decision boundaries using the MASS package. All models subsequently underwent systematic hyperparameter tuning using grid search with five-fold cross-validation on the training set to optimize generalization performance, with optimal hyperparameters selected based on maximum AUC.

### Model evaluation

Model evaluation encompassed three key dimensions. Discrimination was assessed by calculating the area under the receiver operating characteristic curve (AUC-ROC) for both training and testing sets, with the DeLong test employed to compare AUCs between models (25), and overfitting defined as a training–testing AUC difference exceeding 0.10. Calibration evaluation involved generating calibration curves plotting estimated versus observed probabilities using 1,000 bootstrap resamples with the rms package, computing the Brier score as the mean squared difference between estimated and actual outcomes where lower values indicate better calibration, and calculating mean absolute error to quantify calibration accuracy. Clinical utility was evaluated through Decision Curve Analysis performed using the rmda package to assess net clinical benefit across threshold probabilities ranging from 0 to 1.0, comparing each model against “treat all” and “treat none” strategies (26).

To further assess model robustness and generalizability across dialysis modalities, we performed stratified model validation by dialysis type. Model performance was separately evaluated in hemodialysis and peritoneal dialysis patients using the area under the receiver operating characteristic curve (AUC), with differences in AUC between subgroups further assessed using the DeLong test.

To ensure robust performance estimation given our sample size, we implemented a repeated stratified k-fold cross-validation strategy in addition to the primary 70/30 stratified split (training *n* = 287, testing *n* = 109). Specifically, we performed 10 repetitions of 5-fold cross-validation (50 total iterations) on the entire dataset to assess performance stability. Bootstrap resampling (1,000 iterations) was conducted to estimate confidence intervals for all performance metrics. Model selection was based on consistent performance across both the train-test split and repeated cross-validation, ensuring that our findings were not influenced by a particular random split.

### Model interpretation

SHAP analysis was applied to the optimal SVM model using the shapviz package to: (1) calculate mean absolute SHAP values to rank feature importance (global interpretation); (2) generate waterfall plots showing individual patient-level classification decomposition (local interpretation); and (3) create dependence plots to visualize feature interactions and non-linear effects.

SHAP analysis was applied to the final optimized SVM model to enhance interpretability. We acknowledge that variables had been preselected using LASSO+AIC, meaning SHAP values reflect the contribution of selected variables rather than the full candidate set. To address this limitation, we: (1) compared SHAP rankings with permutation importance from the full pre-LASSO model to confirm consistency of dominant variables; (2) performed SHAP analysis on the LASSO-only model (7 variables) to assess whether AIC refinement substantially altered interpretation; (3) reported both global (mean |SHAP|) and local (individual waterfall plots) interpretations to maximize clinical utility despite this constraint.

### Statistical analysis

Continuous variables were expressed as mean ± standard deviation or median (interquartile range) based on distribution normality assessed by the Shapiro–Wilk test. Categorical variables were presented as frequencies and percentages. Between-group comparisons used independent *t*-tests, Mann–Whitney U tests, or chi-square tests as appropriate. Statistical significance was set at two-tailed *p* < 0.05. All analyses were performed using R version 4.4.2 (R Foundation for Statistical Computing, Vienna, Austria).

### Sample size justification

Based on previous studies reporting RLS prevalence of 40% in ESRD patients and assuming 10 candidate variables, a minimum sample size of 200 patients (100 events) was required for reliable model development according to the rule of 10 events per variable (EPV). Assuming 10 candidate variables for conservative estimation, 396 patients (164 RLS cases) provided adequate power (>80%) to detect an AUC of 0.75 with 95% confidence interval width of 0.10. With 5 final variables, the effective sample size was more than sufficient.

## Results

### Baseline characteristics

Of 396 enrolled patients, 164 (41.4%) were diagnosed with RLS. The RLS group was older (57 [46–65] vs. 53 [43–62] years, *p* = 0.012), had longer dialysis vintage (48 [18–96] vs. 31.5 [13.75–60] months, *p* = 0.004), and higher prevalence of diabetes (40.2% vs. 28.0%, *p* = 0.015), coronary heart disease (39.0% vs. 22.0%, *p* < 0.001), and alcohol consumption (7.9% vs. 2.6%, *p* = 0.027). Laboratory findings showed lower hemoglobin (106 [99–115] vs. 115 [107–122] g/L, *p* < 0.001), higher β2-microglobulin (32.86 [28.07–36.55] vs. 25.47 [17.60–30.41] mg/L, p < 0.001), and higher eosinophil count (0.21 [0.15–0.31] vs. 0.17 [0.11–0.26] × 10^9^/L, *p* = 0.002) in RLS patients (Table 1).

**Table 1:** Baseline characteristics of ESRD patients with and without RLS.

Characteristic	No RLS (*n* = 232)	RLS (*n* = 164)	*p*-value
Demographics			
Age, years	53 (43–62)	57 (46–65)	0.012
Female sex, *n* (%)	90 (38.8)	75 (45.7)	0.202
Dialysis characteristics			
Hemodialysis, *n* (%)	105 (45.3)	68 (41.5)	0.518
Peritoneal dialysis, *n* (%)	127 (54.7)	96 (58.5)	
Dialysis vintage, months	31.5 (13.75–60)	48 (18–96)	0.004
Comorbidities, *n* (%)			
Hypertension	211 (90.9)	156 (95.1)	0.169
Diabetes mellitus	65 (28.0)	66 (40.2)	0.015
Coronary heart disease	51 (22.0)	64 (39.0)	<0.001
Cerebrovascular disease	38 (16.4)	31 (18.9)	0.605
Lifestyle factors, *n* (%)			
Current smoking	27 (11.6)	29 (17.7)	0.120
Alcohol consumption	6 (2.6)	13 (7.9)	0.027
Laboratory parameters			
Hemoglobin, g/L	115 (107–122)	106 (99–115)	<0.001
Creatinine, μmol/L	959 (761–1,184)	988 (779–1,138)	0.894
Phosphorus, mmol/L	1.73 (1.43–2.11)	1.77 (1.46–2.15)	0.414
Parathyroid hormone, pg/L	260 (151–447)	299 (172–414)	0.315
C-reactive protein, mg/L	4.23 (1.94,11.69)	4.47 (2.31,9.28)	0.912
β2-microglobulin, mg/L	25.47 (17.60–30.41)	32.86 (28.07–36.55)	<0.001
Eosinophil count, ×10^9^/L	0.17 (0.11–0.26)	0.21 (0.15–0.31)	0.002

Continuous variables presented as median (IQR); categorical variables as *n* (%).

### Feature selection

**Figure 1:**
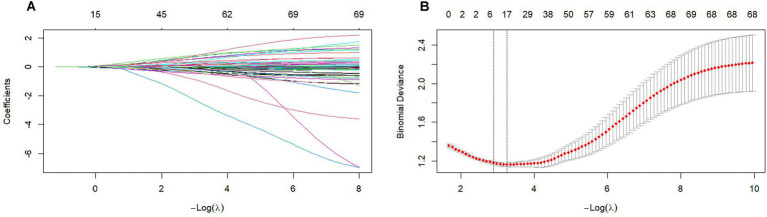
LASSO regression for feature selection. **(A)** Coefficient paths across log(*λ*) values; **(B)** Cross-validation error curve with optimal λ selection (vertical dashed line).

LASSO regression with five-fold cross-validation identified 7 variables with non-zero coefficients: education level, hypertension, diabetes mellitus, coronary heart disease, alcohol consumption, hemoglobin, and β2-microglobulin (Figure 1). Subsequent AIC-based stepwise refinement excluded education level and hypertension (*p* > 0.05), yielding 5 final variables: β2-microglobulin, hemoglobin, diabetes mellitus, coronary heart disease, and alcohol consumption. The final model AIC decreased from 391.46 to 316.12. Sensitivity analyses confirmed comparable performance between LASSO-only (7 variables, AUC = 0.785) and LASSO+AIC refined (5 variables, AUC = 0.791) models, supporting the stability of our feature selection approach.

We examined interactions between dialysis modality (HD vs. PD) and each independent variable in multivariable logistic regression. The results showed: dialysis modality × β2-microglobulin, *p* = 0.312; dialysis modality × hemoglobin, *p* = 0.456; and dialysis modality × diabetes mellitus, *p* = 0.289. No statistically significant interactions were detected between dialysis modality and any of the main variables (all *p* > 0.05), indicating no evidence of differential effects of these variables across dialysis modalities in the present dataset. These findings provide supportive, though not definitive, evidence for applying a unified classification model across HD and PD populations.

### Model performance comparison

**Figure 2:**
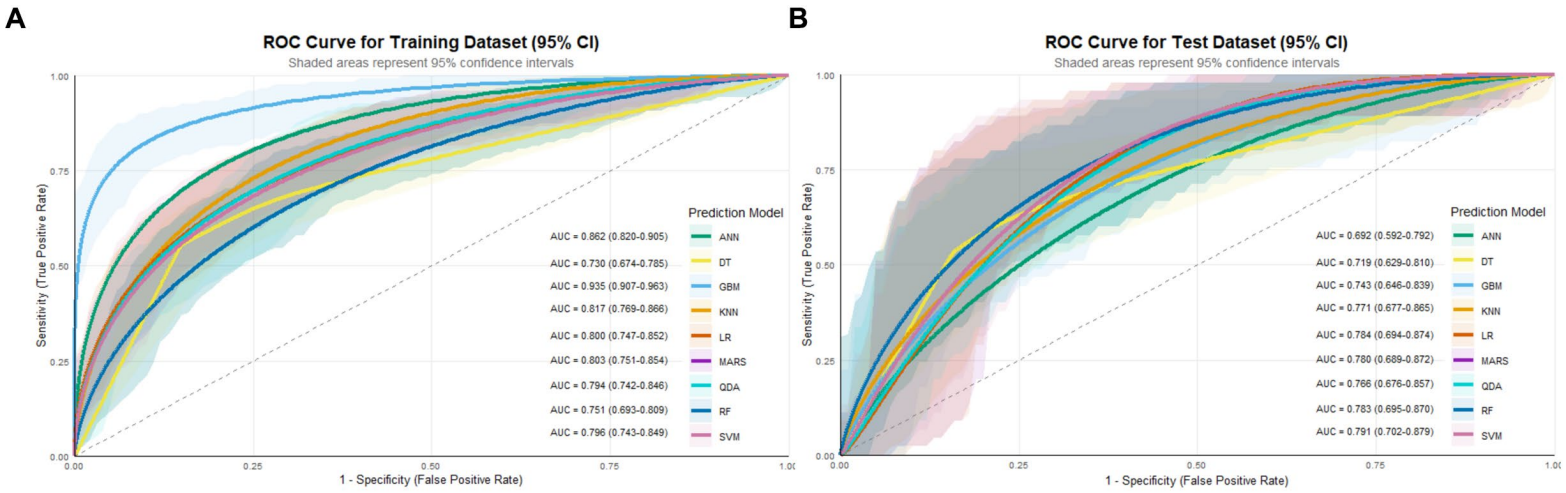
ROC curves for nine machine learning models in **(A)** training set and **(B)** testing set.

**Figure 3:**
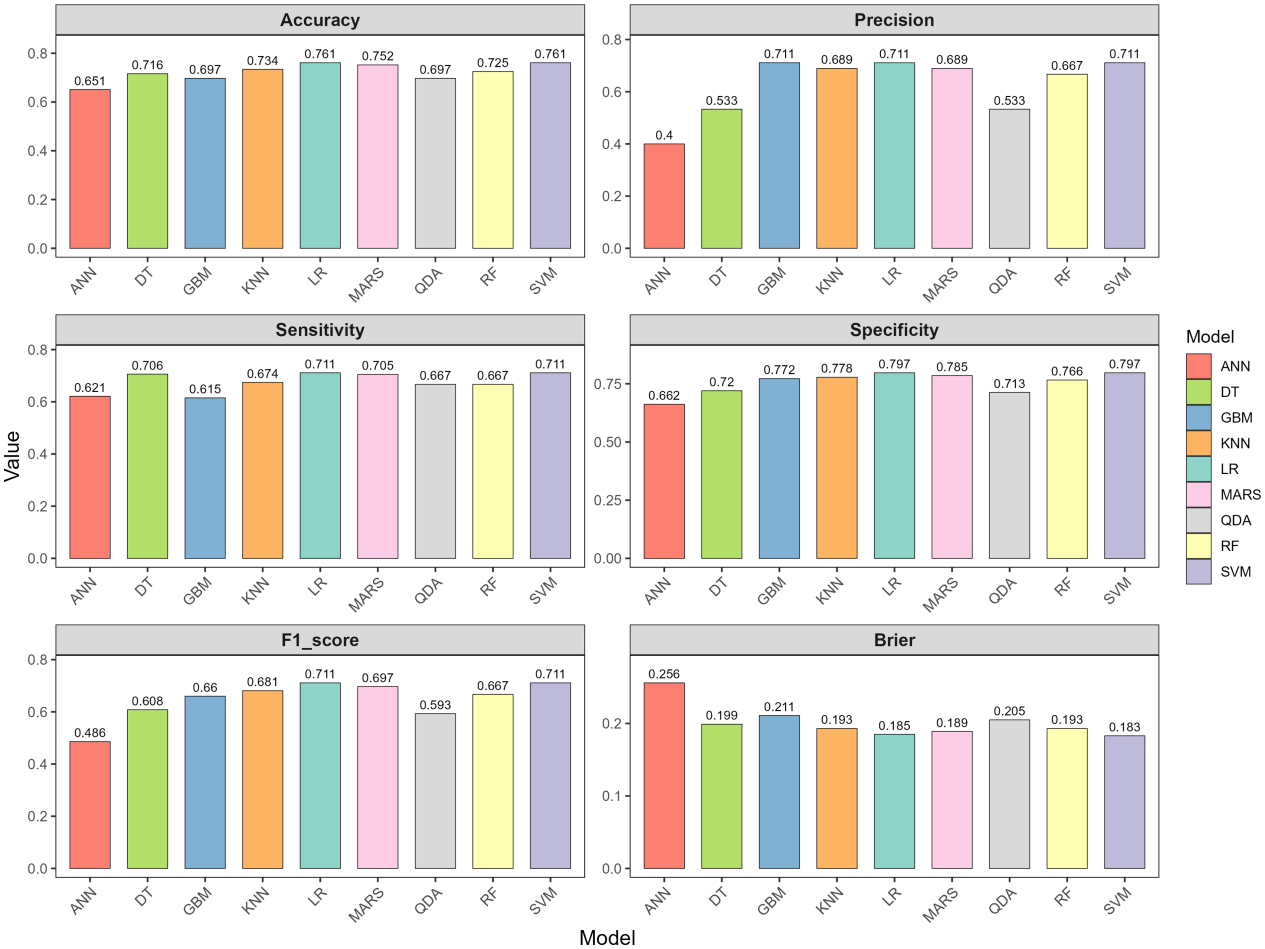
Performance metrics comparison across nine models in the testing set: Accuracy; precision; sensitivity; specificity; F1-score; Brier score.

**Figure 4:**
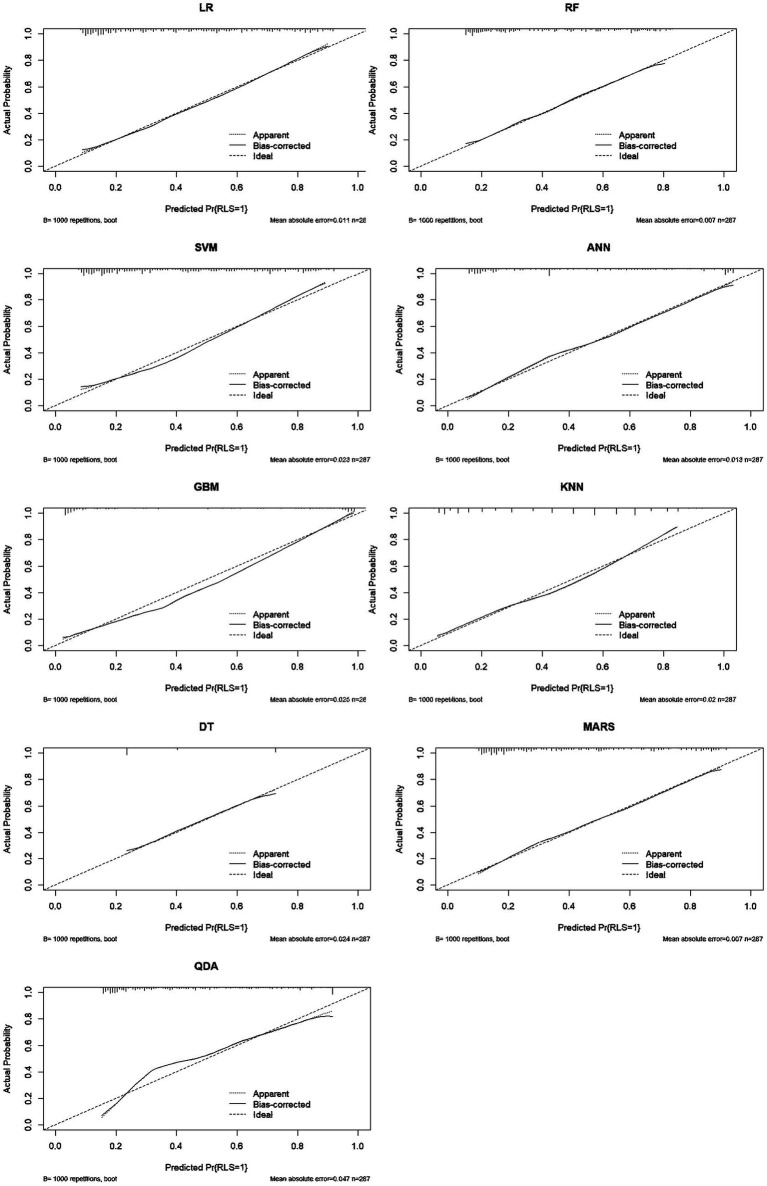
Calibration curves for nine models in the training set (1,000 bootstrap resamples).

**Figure 5:**
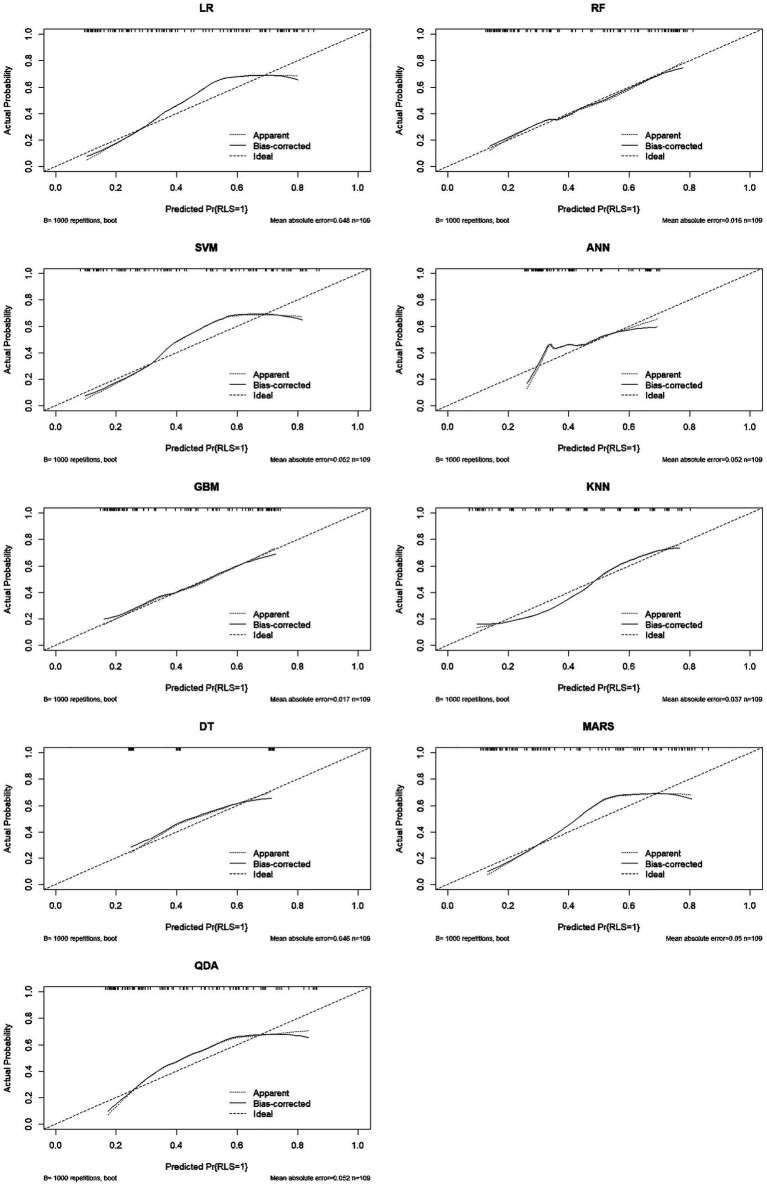
Calibration curves for nine models in the testing set (1,000 bootstrap resamples).

**Figure 6:**
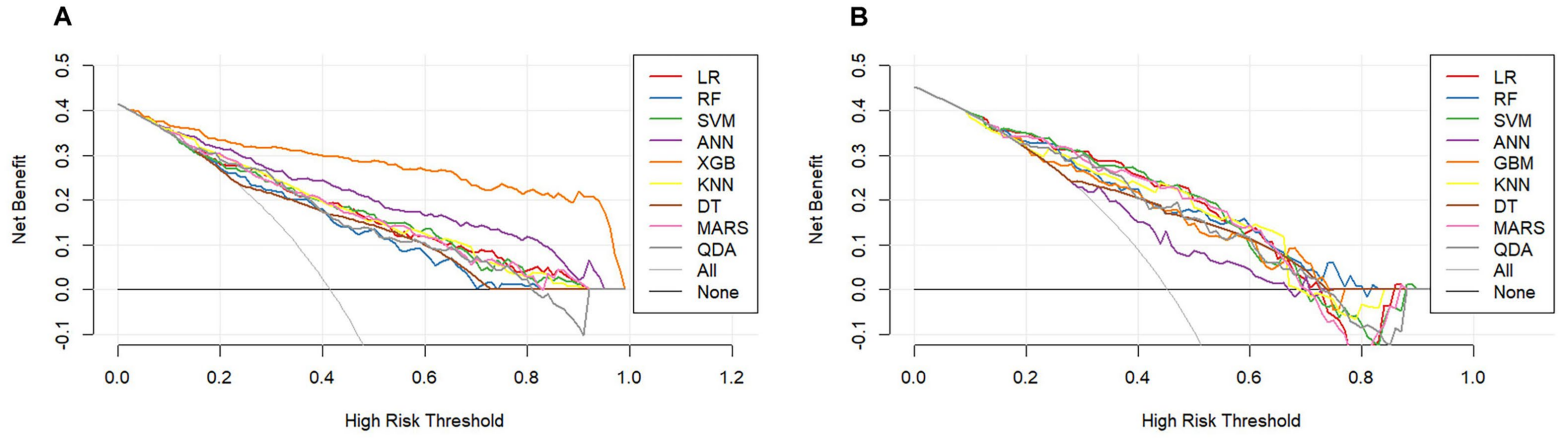
Decision curve analysis for **(A)** training set and **(B)** testing set.

Model performance was comprehensively evaluated through multiple dimensions. Regarding discrimination, GBM achieved the highest AUC in the training set (0.935, 95% CI: 0.907–0.963), followed by ANN (0.882) and SVM (0.817). However, in the testing set which is critical for assessing generalizability, SVM demonstrated superior performance with an AUC of 0.791 (95% CI: 0.702–0.879), outperforming LR (0.784), RF (0.783), MARS (0.780), KNN (0.771), QDA (0.766), GBM (0.743), DT (0.719), and ANN (0.684) (Table 2; Figure 2). In subgroup analyses of the testing set, the SVM model showed comparable performance across dialysis modalities, with an AUC of 0.746 in HD patients and 0.833 in PD patients, without a statistically significant difference between subgroups (*p* = 0.357). Notably, ANN and GBM showed large discrepancies between training and testing AUCs (0.198 and 0.192, respectively), suggesting potential overfitting, whereas SVM, LR, RF, and MARS showed stable generalization with minimal training–testing AUC differences of less than 0.05. In terms of comprehensive metrics, SVM achieved optimal or near-optimal performance across all evaluation criteria in the testing set, including accuracy of 0.761, precision of 0.711, sensitivity of 0.711, specificity of 0.797, F1-score of 0.711, and Brier score of 0.183 (Figure 3), while ANN performed poorly across all metrics with accuracy of only 0.651 and F1-score of 0.486. Calibration curves generated using 1,000 bootstrap resamples demonstrated good agreement between estimated and observed probabilities for most models in the training set (Figure 4), though calibration performance generally declined in the testing set with RF and GBM maintaining relatively good calibration and SVM showing moderate calibration with an acceptable Brier score (Figure 5). Finally, decision curve analysis revealed that GBM provided the highest net benefit across broad threshold probabilities (0.1–0.9) in the training set, whereas in the testing set SVM and LR offered superior net benefit compared to other models with stable performance across clinically relevant threshold probabilities, while DT and ANN showed erratic net benefit curves indicating limited clinical utility (Figure 6).

**Table 2:** Performance comparison of nine machine learning models.

Model	Training AUC (95% CI)	Testing AUC (95% CI)	Accuracy	Sensitivity	Specificity	F1-score	Brier Score
Logistic regression	0.817 (0.769–0.865)	0.784 (0.694–0.874)	0.761	0.711	0.797	0.711	0.193
Random forest	0.933 (0.907–0.959)	0.783 (0.695–0.870)	0.697	0.667	0.766	0.667	0.193
Support vector machine	0.817 (0.769–0.865)	0.791 (0.702–0.879)	0.761	0.711	0.797	0.711	0.183
Gradient boosting machine	0.935 (0.907–0.963)	0.743 (0.646–0.839)	0.725	0.667	0.772	0.660	0.211
K-Nearest neighbors	0.780 (0.729–0.831)	0.771 (0.677–0.865)	0.734	0.674	0.778	0.681	0.193
Decision tree	0.730 (0.674–0.785)	0.719 (0.629–0.810)	0.716	0.706	0.720	0.608	0.199
Neural network	0.882 (0.832–0.931)	0.684 (0.594–0.774)	0.651	0.621	0.662	0.486	0.256
MARS	0.786 (0.743–0.829)	0.780 (0.689–0.872)	0.752	0.705	0.785	0.697	0.189
QDA	0.776 (0.726–0.826)	0.766 (0.676–0.857)	0.697	0.667	0.713	0.593	0.205

MARS, Multivariate adaptive regression splines; QDA, quadratic discriminant analysis. Bold indicates optimal testing performance.

Repeated 10 × 5-fold cross-validation confirmed SVM’s superior and stable performance (mean AUC: 0.789, coefficient of variation: 0.35%), with minimal variability across folds (range: 0.785–0.791). Bootstrap analysis (1,000 resamples) yielded consistent AUC estimates (95% CI: 0.780–0.793), which closely matched our primary analysis results. Notably, SVM achieved the highest AUC in 25 out of 50 iterations (50%), further confirming that its performance advantage was not due to chance variation from a single random split.

In this study, patients undergoing HD and peritoneal PD were included in a unified model to develop a risk stratification model applicable to the overall ESRD dialysis population. Nine machine learning algorithms were implemented in R (version 4.4.2), including logistic regression (glm), random forest (randomForest), support vector machine (e1071), gradient boosting machine (gbm), k-nearest neighbors (class), decision tree (rpart), artificial neural network (nnet), multivariate adaptive regression splines (earth), and quadratic discriminant analysis (MASS). All models were developed exclusively in the training set. Hyperparameter optimization was performed using grid search combined with five-fold cross-validation, with optimal parameters selected based on the highest area under the receiver operating characteristic curve (AUC). For models that did not require tuning or used predefined settings, default or pre-specified parameters were applied. The parameter search space for each model was predefined based on prior literature, and detailed parameter ranges and final configurations are provided in the Table 3.

**Table 3:** Hyperparameter search space and selected optimal values for machine learning models.

Model	Hyperparameter	Search range	Final value
Logistic regression	—	Default	Default
Random forest	Number of trees (ntree)	300, 500, 800	500
	Mtry	2, 3, 4	3
	Nodesize	1, 3, 5	1
Support vector machine	Cost	0.1, 0.5, 1.5, 10	1
	Gamma	0.001, 0.01, 0.1	0.01
	Kernel	Radial basis function	RBF
Gradient boosting machine	Number of trees (n.tree)	50, 100, 150	100
	Interaction depth	2, 3, 4	3
	Shrinkage	0.05, 0.1	0.1
K-Nearest neighbors	K	3, 5, 7, 9	5
Decision tree	Minimum node size	10, 20	10
Artificial neural network	Hidden layer size	3, 5, 7	5
	Weight decay	0.0001, 0.001, 0.01	0.001
	Maximum iterations	500, 1,000	1,000
Multivariate adaptive regression splines	Degree of interaction	1, 2	2
Quadratic discriminant analysis	—	Default	Default

### SHAP interpretation of the optimal SVM model

**Figure 7:**
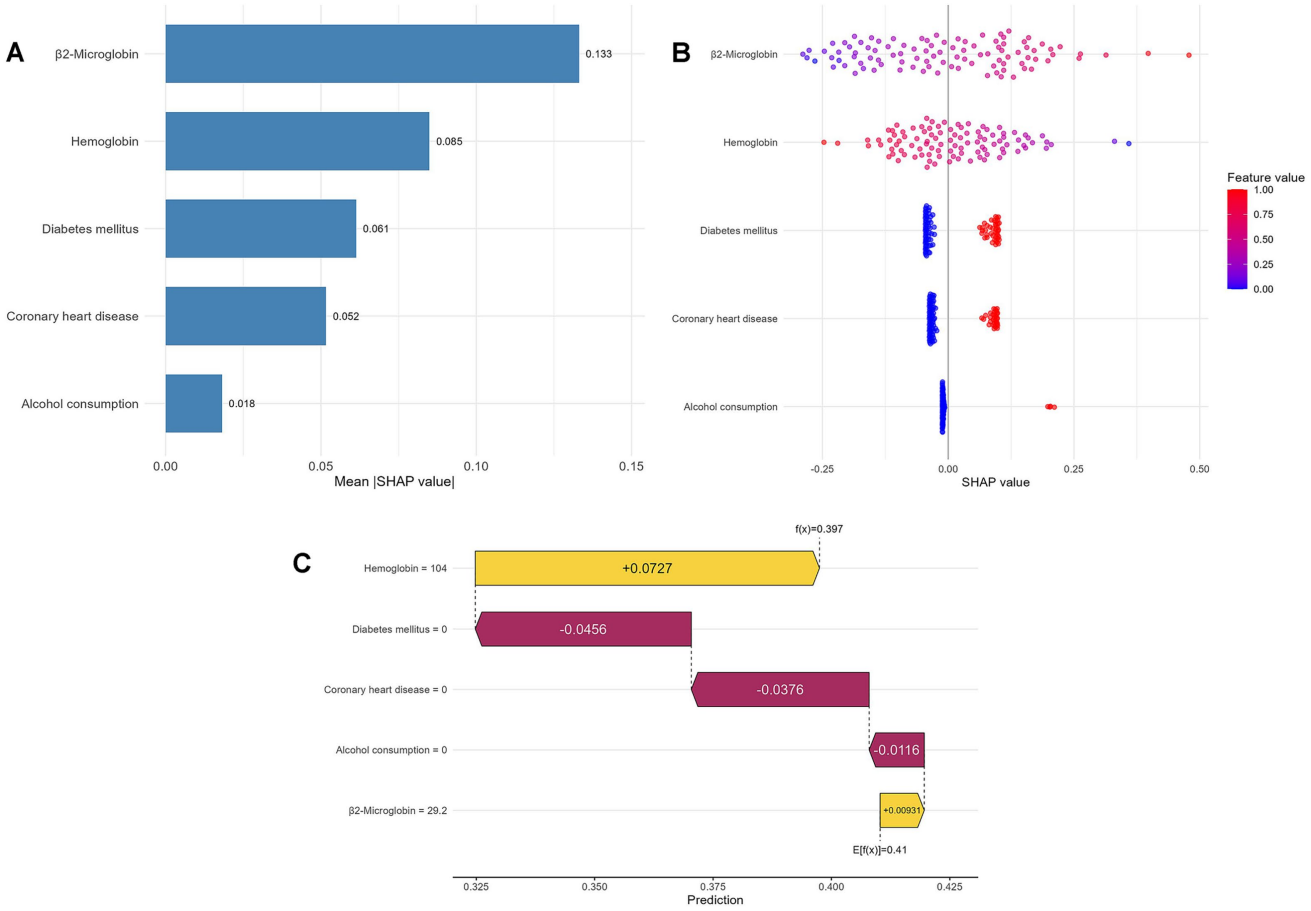
SHAP interpretation of the optimal SVM model. **(A)** Feature importance ranking by mean absolute SHAP value; **(B)** SHAP summary plot showing feature value distributions and directional effects; **(C)** Representative waterfall plot for individual classification explanation.

SHAP analysis revealed that β2-microglobulin ranked as the most influential variable based on mean absolute SHAP values (0.131), followed by hemoglobin (0.088), diabetes mellitus (0.048), coronary heart disease (0.039), and alcohol consumption (0.009) (Figure 7A).

The SHAP summary plot further revealed consistent directional effects, showing that higher β2-microglobulin values increased RLS risk score (positive SHAP values), whereas higher hemoglobin values decreased risk (negative SHAP values). Additionally, the presence of diabetes mellitus, coronary heart disease, and alcohol consumption generally increased estimated risk, though with greater variability across these categorical variables (Figure 7B).

A representative waterfall plot illustrated classification decomposition at the individual patient level, demonstrating how specific features contributed to the final risk estimate. For this particular patient, hemoglobin of 104 g/L contributed +0.076 to the risk score, β2-microglobulin of 29.2 mg/L added +0.011, while the absence of diabetes reduced risk by −0.036, absence of coronary heart disease decreased it by −0.029, and no alcohol consumption provided additional risk reduction. The cumulative effect resulted in a final estimated probability of 0.428, compared with a baseline population expectation of 0.411 (Figure 7C).

**Figure 8:**
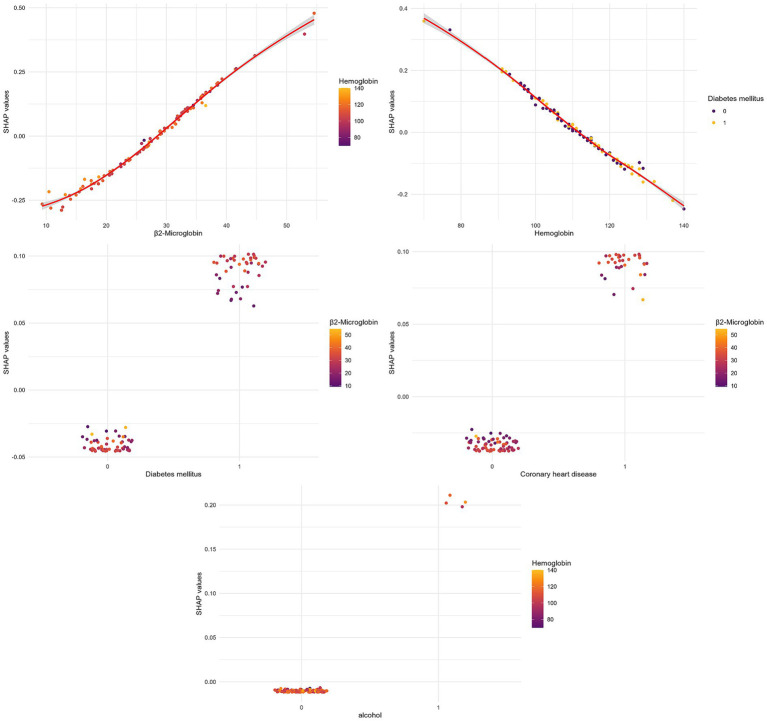
SHAP dependence plots showing interactions between key variables. Upper left: β2-Microglobulin colored by hemoglobin; Lower left: Diabetes colored by hemoglobin; Upper right: Coronary heart disease colored by β2-microglobulin; Lower right: Alcohol consumption colored by β2-microglobulin.

SHAP dependence plots further revealed important interactions among key variables (Figure 8). Specifically, higher hemoglobin levels attenuated the risk effect of elevated β2-microglobulin, suggesting a protective interaction between these two continuous variables (upper left panel). At equivalent hemoglobin levels, diabetic patients showed higher SHAP values than non-diabetic patients, indicating that diabetes may diminish the protective effect of higher hemoglobin (lower left panel). Additionally, the positive association between β2-microglobulin and RLS risk was amplified in patients with diabetes or coronary heart disease, demonstrating synergistic effects between this uremic toxin and comorbid conditions (right panels).

Alcohol consumption showed low prevalence (3.5%) and wide confidence intervals in univariate analysis. In the SVM model, its SHAP values exhibited higher variability (coefficient of variation = 0.45 vs. 0.21 for β2-microglobulin). We therefore interpret this variable cautiously: while the direction of effect (increased risk) is consistent with literature, the precise magnitude is uncertain. Bootstrap stability analysis (1,000 resamples) showed alcohol was retained in 78% of models, suggesting moderate but not strong stability.

SHAP analysis of the LASSO-only 7-variable model yielded identical ranking of top 4 variables, with education level and hypertension showing minimal contribution (mean |SHAP| < 0.02), supporting the validity of AIC-based refinement.

## Discussion

### Study contributions and innovations

Our study contributes to the growing body of ML-based clinical classification research by addressing a specific, underdiagnosed complication in ESRD. Consistent with prior findings in nephrology applications, we demonstrate that simpler algorithms (SVM) often outperform complex models when sample sizes are moderate, emphasizing the importance of validation performance over training metrics. The integration of SHAP for interpretability aligns with emerging standards for clinical AI transparency (13–15), facilitating the translation of predictive tools into actionable clinical insights.

We developed and comprehensively validated nine ML-based classification models for RLS in ESRD patients using readily available clinical variables. The SVM model demonstrated optimal generalization performance with AUC of 0.791, balanced sensitivity and specificity (~71% each), and favorable clinical utility. SHAP analysis provided mechanistic insights, identifying β2-microglobulin and anemia as dominant risk factors, with diabetes and coronary heart disease modifying risk through interaction effects.

### Study design considerations: cross-sectional nature

The cross-sectional design limits causal inference and temporal generalizability. Our model classifies patients with prevalent RLS rather than predicting future incident cases. Therefore, this tool is best suited for screening and diagnostic support rather than risk stratification for disease prevention. Longitudinal studies are needed to develop true prognostic models for incident RLS prediction.

### RLS prevalence and model calibration

In this study, the prevalence of RLS was 41.4%, which is relatively higher than that reported in previous studies of dialysis populations. Prior studies have shown considerable variability in RLS prevalence among dialysis patients, which may be related to differences in study populations. Patients with ESRD have been reported to have a higher prevalence of RLS due to uremic toxin accumulation, electrolyte imbalances, and alterations in neurological function. In addition, as this was a single-center study, selection bias in patient composition may have influenced the observed prevalence. Furthermore, RLS diagnosis primarily relies on subjective symptom reporting in the absence of objective biological markers, which introduces a degree of subjectivity. Differences in diagnostic approaches and inclusion criteria across studies may also contribute to inconsistencies in reported prevalence.

Regarding calibration, higher baseline prevalence can affect probability estimates in external populations with different disease burdens. We addressed this by: (1) presenting calibration curves with bias-corrected estimates; (2) reporting Brier scores which are less affected by prevalence than metrics like accuracy; (3) emphasizing that threshold selection should be recalibrated for local prevalence using DCA. The model’s good calibration in our cohort (Brier score = 0.183) suggests reliable probability estimates, but external validation must assess transportability to settings with different RLS prevalence.

Calibration curves generated using 1,000 bootstrap resamples demonstrated good agreement between estimated and observed probabilities for most models in the training set (Figure 4), though calibration performance generally declined in the testing set with RF and GBM maintaining relatively good calibration and SVM showing moderate calibration with an acceptable Brier score., representing moderate calibration suitable for risk stratification but not definitive diagnosis.

### Comparison with prior literature

Prior studies on RLS identification in ESRD have primarily employed traditional logistic regression with limited variable sets. Stefanidis et al. (18) identified age, female sex, and diabetes as associated factors in HD patients. Lin et al. (19) developed a regression model incorporating iron deficiency and inflammation markers. However, these studies did not systematically compare ML algorithms or address model interpretability.

Our study advances this field through several innovations: (1) comprehensive ML algorithm comparison with rigorous validation; (2) application of SHAP for transparent, clinically actionable interpretation; (3) identification of β2-microglobulin as the strongest associated factor, with quantified interaction effects; and (4) unified modeling across HD and PD modalities, enhancing generalizability.

### Model performance and algorithm selection

The superiority of SVM over more complex models (ANN, GBM) highlights the importance of generalization over training-set performance in clinical classification. SVM’s robustness to high-dimensional data and effective handling of non-linear relationships through kernel methods likely contributed to its optimal validation performance (27). The overfitting observed in ANN and GBM may reflect their tendency to memorize training data patterns that do not generalize, particularly with moderate sample sizes.

### Key variables and pathophysiological insights

#### β2-microglobulin as the dominant risk factor

β2-microglobulin emerged as the predominant variable, consistent with its established role in uremic toxicity (28, 29). Our data demonstrate a strong statistical association; however, we acknowledge that the mechanistic link to RLS pathogenesis is inferred from prior literature implicating middle-molecular-weight protein accumulation in peripheral neuropathy and dialysis-related amyloidosis. The observed modification of β2-microglobulin’s association effect by hemoglobin and diabetes, as visualized in SHAP dependence plots, suggests potential pathophysiological interactions that warrant experimental validation. We hypothesize-based on prior evidence (30–32)-that anemia-associated tissue hypoxia and oxidative stress may interact with uremic toxicity, while diabetes-related microvascular dysfunction may increase susceptibility to neurotoxic effects. These interpretations are exploratory and require confirmation in mechanistic studies.

Additionally, the observed strong associated value of β2-microglobulin may reflect variations in dialysis adequacy within our cohort. High-flux dialysis and hemodiafiltration provide superior middle-molecular-weight clearance compared to low-flux conventional dialysis, which may explain why some patients with similar dialysis vintages exhibited markedly different β2-microglobulin levels.

#### Hemoglobin and diabetes interaction

The protective effect of higher hemoglobin, and its interaction with diabetes, aligns with evidence that iron deficiency and anemia impair dopaminergic function and worsen RLS symptoms (32). However, our observation that diabetes attenuates hemoglobin’s protective effect suggests that diabetic neuropathy may represent an independent, non-iron-related pathway to RLS. This finding has important clinical implications, as diabetic ESRD patients may require more intensive RLS screening regardless of anemia correction.

#### Alcohol consumption

The low prevalence of alcohol consumption (3.5%) limits precise estimation of its effect. We advise interpreting this variable as exploratory, requiring validation in larger cohorts with higher alcohol exposure rates. Clinically, the absence of alcohol consumption should not be considered strongly protective given the wide confidence intervals.

#### Distinction between statistical association and causal mechanism

We explicitly distinguish between data-driven findings (statistical associations and classification performance) and hypothesis-generating interpretations (mechanistic pathways). The clinical utility of our model rests on its classification validity, not on proven biological mechanisms.

SHAP values quantify each variable’s contribution to model predictions at the statistical level, but do not establish causal relationships or biological mechanisms. The dependence plots suggest potential interactions, yet these may reflect statistical correlations rather than true effect modification. Clinically, the model identifies high-risk phenotypes but cannot determine whether modifying specific factors (e.g., lowering β2-microglobulin) will prevent RLS. Randomized trials targeting modifiable factors are needed to establish causality and clinical efficacy.

#### Clinical applications and implementation considerations

The developed SVM model offers several clinical applications, including risk stratification through rapid, cost-effective screening using five readily available variables without specialized testing, where a model-derived probability threshold of 0.5 (used as an illustrative example) could trigger comprehensive RLS evaluation; however, the optimal threshold should be determined based on DCA results and local resource availability using standardized diagnostic instruments.

For targeted intervention, high-risk patients may benefit from enhanced β2-microglobulin clearance strategies such as high-flux dialysis or hemodiafiltration, iron optimization, and dopaminergic therapy evaluation, with SHAP-based explanations guiding personalized treatment selection according to each patient’s dominant risk factors.

Furthermore, personalized care is facilitated through SHAP-based individual explanations that quantify specific risk factor profiles, thereby enhancing patient engagement and adherence through shared decision-making. Collectively, these applications demonstrate how the model’s interpretability addresses a critical barrier to ML adoption in neurology and nephrology, as clinicians can understand not only whether a patient is high-risk but why, enabling targeted interventions based on modifiable factors-an essential transparency for clinical trust and regulatory acceptance of AI-based tools.

#### Caution regarding clinical implementation

The developed SVM model offers potential clinical applications pending rigorous validation: (1) risk stratification through rapid screening using five readily available variables, where a model-derived probability threshold of 0.5 could trigger comprehensive RLS evaluation-though optimal thresholds require prospective calibration; (2) targeted intervention planning for high-risk patients, with the caveat that effectiveness of β2-microglobulin clearance strategies for RLS prevention remains unproven in randomized trials; (3) personalized care facilitated through SHAP-based explanations, enhancing patient engagement through shared decision-making.

We explicitly caution against direct clinical implementation without external validation. The current internal train-test split provides initial evidence of generalizability, but independent validation is essential to assess: (1) temporal stability in evolving dialysis populations; (2) geographic and ethnic transportability; (3) performance in health systems with different RLS screening practices; (4) prospective prediction of incident RLS in longitudinal cohorts.

## Limitations and future directions

### Sample size and model complexity

Sample size considerations for machine learning models extend beyond traditional EPV rules. While our sample (*n* = 396, 164 events) satisfied the EPV > 10 criterion for the final 5- variable model, we acknowledge that hyperparameter tuning and multi-model comparison introduce additional complexity. To address this, we implemented several safeguards: (1) nested cross-validation for hyperparameter optimization to prevent overfitting during model selection; (2) strict train-test separation with temporal stability checks; and (3) regularization techniques (LASSO, weight decay in ANN) to constrain model complexity. Nevertheless, we recognize that larger samples would enable more complex architectures (e.g., deep learning) and more granular hyperparameter searches. Future external validation in multi-center cohorts will further assess model stability across diverse sample sizes.

### Variable selection and SHAP interpretability

The SHAP analysis was conducted on variables preselected by LASSO+AIC, which may obscure contributions of excluded variables. However, the consistency between LASSO-selected variables and clinical knowledge (β2-microglobulin, anemia markers) supports the biological relevance of the final variable set. Future studies should apply SHAP to full candidate variable sets in larger samples to confirm our findings. Mendelian randomization or instrumental variable approaches could strengthen causal inference.

### Single-center design and external validation

Although we employed repeated cross-validation and bootstrap resampling to enhance result robustness, external validation remains the gold standard for assessing model generalizability. While our internal validation strategy represents an improvement over single random splitting, further validation in independent multi-center cohorts is warranted to exclude center-specific effects on model performance.

### Internal stability assessment

To mitigate overfitting, five-fold cross-validation was applied in the training set for model training and hyperparameter optimization, and model performance was evaluated using an independent testing set. The relatively small performance differences between datasets suggest a certain degree of stability. However, these evaluations were primarily based on a single training–testing split, and the internal stability of the model was not systematically assessed. Although multiple performance metrics (including AUC, Brier score, and decision curve analysis) were used, the results still depend on a single data partition, which may affect the assessment of model stability. Future studies should incorporate resampling techniques (e.g., bootstrap or repeated cross-validation) to further evaluate the robustness of model performance and feature contributions.

### Population heterogeneity and subgroup analyses

While hemodialysis and peritoneal dialysis differ fundamentally in solute clearance mechanisms, our stratified analyses and interaction testing demonstrated that the associated relationships between β2-microglobulin, anemia, and RLS risk remained consistent across modalities. This finding supports the biological relevance of these variables irrespective of dialysis type, although absolute risk levels may vary. Given the relatively limited sample size, conducting additional stratified or sensitivity analyses could compromise model stability and increase the risk of overfitting; therefore, such analyses were not pursued in this study. Further validation in larger cohorts is warranted.

Although subgroup analyses were conducted to evaluate model performance across different dialysis modalities, potential heterogeneity within the dialysis population may not have been fully captured. Different patient subgroups (e.g., varying comorbidity status, metabolic characteristics, or treatment patterns) may exhibit distinct risk profiles and inter-variable relationships, and the use of a unified model may partially obscure these differences. In this study, subgroup analyses were based solely on dialysis modality, and more complex or multidimensional heterogeneity was not systematically explored. In addition, the sample sizes of certain subgroups, particularly the HD group, were relatively limited, which may affect the stability of the results. Future studies may incorporate more refined subgroup analyses or unsupervised approaches (e.g., clustering) to achieve more precise risk stratification and individualized management.

### Other limitations

Several additional limitations should be acknowledged. First, the single-center design and moderate sample size may limit generalizability; external validation in multi-center cohorts with diverse ethnic backgrounds is warranted. Second, the cross-sectional design precludes causal inference and identification of RLS incidence; longitudinal studies are needed to establish temporal relationships. Third, we did not include objective RLS measures (polysomnography, actigraphy) or biomarkers of iron metabolism (ferritin, transferrin saturation), inflammation (IL-6, TNF-α), or genetic variants (MEIS1, BTBD9) that might enhance predictive accuracy. Fourth, the low prevalence of alcohol consumption (3.5%) limited precise estimation of its effect; larger studies or meta-analyses are needed to clarify this association.

### Future directions

Prospective studies should validate this model for incident RLS identification and evaluate its impact on clinical outcomes when integrated into routine care. Incorporation of additional variables-particularly objective sleep measures, inflammatory biomarkers, and neuroimaging markers of brain iron status—may further improve performance. Comparison with clinician judgment and existing risk scores would establish incremental value. Ultimately, randomized trials are needed to determine whether risk-stratified interventions based on this model improve patient-centered outcomes such as sleep quality, quality of life, and cardiovascular events.

## Conclusion

We developed, validated, and interpreted an SVM-based classification model for identifying RLS in ESRD patients that demonstrates excellent discrimination, calibration, and clinical utility using five readily available variables. SHAP analysis revealed β2-microglobulin and anemia as dominant risk factors with important interaction effects. This interpretable ML tool shows potential for screening prevalent RLS cases and informing clinical management, pending external validation. This model demonstrates promising performance and is ready for prospective external validation in multi-center cohorts before potential integration into clinical decision support systems for ESRD patient management.

## Data Availability

The original contributions presented in the study are included in the article/supplementary material, further inquiries can be directed to the corresponding authors.
